# Michigan State Board of Health

**Published:** 1880-11

**Authors:** 


### Society Reports.

### ARTICLE VIII.

MICHIGAN STATE BOARD OF HEALTH. Reported for THE CHICAGO MEDICAL JOURNAL AND EXAMINER.

The regular quarterly meeting of this board was held at its office in the State capitol at Lansing on Tuesday, October 12th, 1880. The following members were present during the meeting: Prof. E. A. Strong, of Grand Rapids; Hon. Le Roy Parker, of Flint; Rev. D. C. Jacokes, of Pontiac; H. F. Lyster, M.D., of Detroit; J. H. Kellogg, M D., of Battle Creek; and Henry B. Baker, M.D., secretary.

### IMPURE WATER.

Dr. Kellogg reported the completion of his paper on contamination of water by decaying wood, and mentioned in that connection some observations of his in regard to ice being contaminated by decaying sawdust and other impurities. He showed the fallacy of the popular belief that ice freezes pure, and said that it incloses all organic impurities that float. He described a water-cooler which was designed to avoid contamination of the water by the ice, as would happen if the ice were placed directly in the water. A cylinder containing ice was placed in the center of the cooler allowing the water to come in contact with this cold cylinder without touching the ice. He also reported progress in studies relative to the work of the new committee to which he was appointed, "the relations of preventable sickness to taxation." Dr. Baker made a report of the

### WORK IN THE SECRETARY'S OFFICE

during the past quarter, which showed the distribution of a large number of annual reports and other documents to officers of local boards of health and other persons. Heretofore documents have usually been sent to the county clerks for distribution to local officers, but having seen that it might be as difficult for some persons to get them from the county clerks' offices as from Lansing, the secretary sent a circular letter to presidents of villages, asking them whether they wished them sent to county clerks, or if they would pay the express charges if sent to them direct. Of 102 replies, seventy-three desired the packages sent direct, twenty-nine wished them sent to the county clerks, and of the latter, many now lived at or near county seats. Many of these officers expressed great interest in the information contained in the documents of the State board. From evidence collected at Lansing, it would seem that the documents issued by the State board of health are in greater demand than any State documents, with the exception of the reports of the State board of agriculture and State pomological society.

### REGULATION OF MEDICAL PRACTICE.

The secretary stated that in response to communications relative to the proposed regulation of medical practice, he had prepared a paper and a form for a bill. He submitted an outline of it to the board. He had done this partly because he feared the State board of health would be made the examining board, and its usefulness for other important work impaired.

Later in the session Dr. Lyster spoke on the same subject, and the following resolutions were adopted by the board:

*Resolved*, That there should be required of all who are to begin the practice of medicine in this State an examination as to their qualifications.

*Resolved*, That such examinations by the State should be restricted to questions in demonstrable knowledge as distinguished from questions of mere opinion.

*Resolved*, That as a public health measure, a committee of three be appointed to prepare and report, at the next meeting of the board, a plan for furthering the objects stated in the preceding resolutions.

Drs. Lyster and Baker, and Rev. Dr. Jacokes, were appointed such committee.

### THE ANNUAL REPORT OF THE SECRETARY

relative to property received and disposed of during the fiscal year ending September 30, 1880, showed the purchase and placing of meteorological instruments in different parts of the State, the addition of 414 books and pamphlets to the library of the board, the receipt of weekly and monthly mortality statements from the principal cities in the United States and some foreign countries, the distribution of similar information respecting Lansing and the State; the detailed expenditures of the office, which are classified as follows:

Expenses of members attending meetings, $205.65; instruments and books, $147.11; paper, stationery, etc., $192.51; postage for the office, $581.90; postage by members, $16.30; printing and binding, $389.27; secretary, $2,000; miscellaneous (which includes telegrams, express, freight, etc.), $120.39; making a total expenditure for the fiscal year of $3,653.13.

### EXAMINATIONS IN SANITARY SCIENCE.

The secretary reported that Dr. M. Veenboer, of Grand Rapids, and Henry B. Baker, M.D., of Lansing, the applicants for examination in sanitary science by this board, July 14, both passed the examination, and the board had since voted to grant them certificates. It was voted to publish the questions asked these candidates, in the report of the board for 1880. The secretary reported that, in accordance with instructions from the board, he had prepared a list of books valuable for reference and study by candidates for the examinations in sanitary science, and it was voted to print the list in the annual report for 1880.

### OZONE.

An interesting paper by J. Mulvany, M.D., of the British navy, giving the results of ozone observations conducted in various parts of the world, was presented, accepted with thanks, and ordered published in the annual report. The paper was read before the Meteorological Society, London, England, but not yet published.

### REMOVAL OF A SMALL-POX CORPSE.

The secretary presented a letter describing the method of re-interment, under the direction of the health officer of Lansing, of the body of a person who had died of small-pox.

### SANITARY CONVENTIONS.

It was voted to hold two sanitary conventions for the reading of papers, discussion of sanitary topics, and the exhibition of sanitary appliances, during the coming winter. Rev. Dr. Jacokes and Dr. Baker were appointed a committee to receive invitations and make arrangements for the conventions. Persons desiring a convention at any place, may correspond with either member of the above committee.

Prof. Strong said the convention at Grand Rapids last winter had greatly stimulated public health work in that city.

The secretary presented an invitation to the international medical congress to be held in London, August, 1881.

Dr. Jacokes presented a drawing and description of plan for introducing fresh air to be warmed by a coal stove in the room.

The secretary was directed to investigate the hog cholera now prevailing in the southwestern part of this State, and find, if possible, any relation between that and any sickness in the human species.

Prof. Strong, the new member, was assigned to work on the committees on the "relations of schools to health," and on "the relations of climate to health."

Dr. Baker presented specimens of pine infected with a fungus which had completely destroyed the floors of several rooms, constructed of that wood, in a new building. The fungus seemed to grow most where the floor was covered, as with oil-cloth, or by boxes resting on the floor: and in one room the decayed floor corresponded with the portion not exposed to light, though that case may be explained by a greater amount of moisture in that part of the room, because of dampness underneath. The odor in the room was that mouldy or musty odor not infrequently met with in close rooms. It caused frontal headache, and a person engaged in repairing the floor had spells of sneezing on two occasions some months apart, while thus employed.

The secretary presented communications from E. P. Christian, M.D., of Wyandotte, relative to diphtheria, etc., and he was instructed to use them in the annual report.

A design for an official seal for the board was presented by Dr. Baker, and adopted.

Dr. Henry B. Baker was appointed a delegate to the meeting of the American Public Health Association at New Orleans, in December.

Auditing of bills and other routine work was accomplished during the day. The next regular meeting of the board will be on January 11th, 1881.

---

AN ANECDOTE OF LATHAM.—Dr. George Johnson, F.R.S., Physician to and Professor of Clinical Medicine at King's College Hospital, in the *Medical Times and Gazette* of October 9th, tells the following: "The late Dr. Latham related the following anecdote to Sir Thomas Watson, from whom I heard it: Dr. Latham, as many of you are aware, was a very eminent, learned and accomplished physician of St. Bartholomew's Hospital, but he had published more on the diseases of the heart and lungs than upon any other subject. A patient of his who had recently recovered from some pulmonary affection, one day said to him, 'I feel that as regards my lungs I am quite well, and now I think of going to consult Dr. Watson about my general health.' To which Dr. Latham replied, 'Yes, I see; in your estimation Dr. Watson is an architect, and me, I suppose, you look upon as a bell-hanger.'"—*Louisville Medical News.*

SYMMETRICAL NEURALGIA IN DIABETES.—Dr. J. Worms read a paper on this subject before the Académie de Médecine, in which the following points are brought forward: 1. In a great proportion of cases of diabetes mellitus there exists a bilateral symmetrical neuralgia; 2. The dental and sciatic nerves seem to be the favorite points of attack; 3. Ordinary anti-neuralgics (quinine, morphine, the bromides, chloral, etc.) do not affect these neuralgias as they do others; 4. The neuralgia is very severe, and is increased or diminished in proportion as the glycosuria rises or falls.

